# Editorial: thematic issue on modulating the environment with microbes

**DOI:** 10.1093/femsmc/xtae021

**Published:** 2024-07-27

**Authors:** Utkarsh Sood, Gauri Garg, Rup Lal

**Affiliations:** Department of Zoology, Kirori Mal College, University of Delhi, Delhi 110007, India; Department of Zoology, Kirori Mal College, University of Delhi, Delhi 110007, India; Acharya Narendra Dev College, University of Delhi, New Delhi 110019, India

## Abstract

The significance of heme to Enterococcus faecalis is reviewed while also identifying the prevalence of hemoproteins throughout the enterococci and highlighting gaps in knowledge in enterococcal mechanisms of heme homeostasis.

The advancements in sequencing technology and computational biology have revolutionized microbiology in recent years, for sustaining life on this planet Earth and harnessing the potential of microbes for various applications beneficial to humanity. In particular, these advances have revealed that microbes play diverse and indispensable roles in human welfare from food production to environmental sustainability, healthcare, and industrial processes. Understanding and harnessing the capabilities of these microscopic organisms are key to addressing global challenges and improving the quality of life on the planet Earth. In addition, the knowledge generated from *in silico* studies has now enabled scientists to exploit with ease the potential of microbes in the generation of nitrogen, fixation of the oceanic CO_2_, production of oxygen, production of dairy products, alcoholic drinks, organic acids, enzymes, steroids, in sewage and waste treatment, fertility of soil, production of vitamins, antibiotics, vaccines, and antiviral compounds. Recently, the extraordinary potential of microbes has been proposed to achieve the majority of the 17 sustainable development goals by the United Nations (Anand et al. 2023).

The thematic issue from *FEMS Microbes*, titled “Modulating the environment with microbes,” is thus a step to highlight the vast potential of microbes, particularly the knowledge for their exploitation.

In this special thematic issue, there are six research articles and one review paper that have been published and highlight the critical ecological roles of microbes in diverse settings of normal and extreme environments like hot springs, hypersaline environments, deep-sea hydrothermal vents, acidic mine drainages, and polar ice caps. This demonstrates their remarkable adaptability and importance in biogeochemical cycles, nutrient recycling, and supporting ecosystem functions. This issue also covers articles that reflect the key role of amplicon and whole metagenomics sequencing in the identification of these microbes and also give insights into the functions performed by these microorganisms. For example, Balcha et al. (2024) analyzed metagenomic sequences to find biosynthetic gene clusters (BGCs) from Lake Afdera, Ethiopia. It is a hypersaline lake, whose microbiome was predominantly dominated by two bacterial genera *Acinetobacter* (18.6%) and *Pseudomonas* (11.8%). The study identified 94 distinct BGCs, which encode secondary metabolites with potential pharmaceutical applications (such as polyketide synthase and nonribosomal peptide synthase (NRPs)) and roles in adapting to extreme environments (such as bacteriocins and ectoine). NRPs (20.6%) and bacteriocins (10.6%) were particularly abundant. The study also predicted gene clusters that help microbes resist toxic metals and cope with oxidative and osmotic stress. The application of computational tools thus reflects that Lake Afdera is a rich source of biologically significant BGCs essential for the existence and adaptation of extremophiles.

In addition to the critical roles played by bacteria, ciliates (eukaryotic microbes) have been shown to play vital roles in nutrient recycling, microbial loops, and ecosystem maintenance. Abraham et al. (2024) elucidated the taxonomic diversity of freshwater ciliates at three sites in Delhi, India. They used a DNA metabarcoding approach (18S-V4 barcode) and reported *Oligohymenophorea, Prostomatea*, and *Spirotrichea* as the dominant genera of ciliates at the three freshwater sites. As ciliates are sensitive to changes in their environment, the native genera can be used as potential bioindicators for understanding climatic variations, conducting diversity assessments and monitoring environmental changes across diverse regions.

The application of microbes as biocontrol agents and an alternative to xenobiotic chemicals has great prospects. The use of microbes minimizes the harsh effects of chemical fungicides in a sustainable way. Bhagat and Vakhlu (2024) reported the use of *Bacillus* sp. strain D5 (Bar D5; native biocontrol agent) against *Fusarium oxsporum* R1 (Fox R1) in the saffron corm. Bar D5 inhibited the growth of Fox R1 as observed *in vitro* and *in planta* (saffron corm) by real-time imaging. They undertook analyses to understand the microbial interactions and mechanism of action leading to the inhibition of pathogens at the molecular level. Transcriptome analysis showed that in corms treated with Bar D5 and infected with Fox R1, genes related to growth, metabolism of carbon, and synthesis of the cell wall and membrane were significantly downregulated compared to corms infected only with Fox R1.

Recent studies using omics approaches have revealed that most microbes are friends of mankind. However, the spread of antimicrobial resistance (AMR) in the environment is one of the most critical global coercions. Waste and sludge from pharmaceutical industries and dumpsites are reported to inhabit bacterial populations exhibiting resistance to antimicrobial agents. Patra et al. (2024) analysed sludge samples from sewage treatment plant (STP) to evaluate the antibiotic-resistant bacterial population. They demonstrated that STP sludge contained varied multidrug-resistant bacterial populations and metagenome sequences revealed the presence of many AMR genes and mobile genetic elements. They could also isolate bacterial strains (*n* = 6) that exhibited resistance to 13 antibiotics and had high metal tolerance. Many of the isolated strains were reported to endure high concentrations of vancomycin and clarithromycin, highlighting the severity of antibiotic resistance spread. This research offers new insights into microbial physiology and guidance on preventing the development and spread of AMR. The use of STP sludge could introduce resistance genes into agroecosystems, necessitating further investigation and the development of strategies to combat AMR spread and improve STP technologies to remove emerging contaminants.

In the past, it was difficult to investigate and explore the vast potential of actinobacteria for the production of secondary metabolites most of which have antibacterial activity. Choksket et al. (2023) isolated an antimicrobial-producing Gram-positive, nonmotile, aerobic, and filamentous actinobacterial strain SKN60^T^ from the soil. Genome sequence analysis identified a thiopeptide biosynthetic gene cluster (BGC) with a 94% similarity to the berninamycin cluster from *S. bernensis* UC5144. The observed mass of the reported thiopeptide matched that of berninamycin. Importantly, strain SKN60T was reported as the only known carrier of the berninamycin BGC among all the phylogenetic neighbors in the study.

Actinomycetes strains produce mycelial pellets when subjected to submerged cultivation, which is undesirable in fermentation because it limits mass transfer, alters medium rheology, and impacts secondary metabolite production. Kumar et al. (2023) studied factors regulating mycelial morphology and the mechanism of pellet formation in *Streptomyces toxytricini* strain NRRL B-5426 using microscopic and proteomic analysis. Microscopic analysis showed that hyphae had a bud-like structure near the apical tip that follows an ordered growth pattern, leading to pellet formation during shake flask inoculation. Proteomic studies have identified low molecular weight proteins (20–95 kDa) as key players in apical growth and hyphal branching, contributing to mycelial network formation and potentially regulating pellet morphology. Their research can be applied to develop a protocol to reduce pellet formation, and hence increasing the yield of the produced secondary metabolite.

Further, there is a huge demand for other important secondary metabolites produced by bacteria, yeast, and fungi. One of them is biologically produced surfactants that have been regarded as the best alternative to chemical surfactants (Sood et al. 2020). Kumari et al. (2023) compiled and reviewed green active biosurfactants commonly originating from contaminated sites, natural environments, and marine ecosystems. They described the importance of biosurfactants for different domains of environmental compatibility, biodegradability, stability at a wide pH range, and low toxicity. The review also had up-to-date information describing the various techniques used for screening the next generation of microbial biosurfactants. They also reported the recent advancements in emerging applications of biosurfactants in pharmaceuticals, cosmetics, food, and agricultural industries. The review also explored solutions to mitigate the high costs of production of biosurfactants by using low-cost substrate media formulations, the latest solvent-free foam fractionation methods for extraction, and gravitational techniques that can be implemented in large-scale biosurfactant production.

In brief, the special issue on modulating the environment with microbes highlights the scope of omics to explore the tremendous potential of microbes hitherto unknown for the production of several biomolecules, particularly for human welfare. In addition, by using recently emerging tools of computational biology, we can exploit this power of microbes to address several environmental problems and pave the way for a healthier planet for the betterment of human beings.
